# Identification of SERPINA1 as single marker for papillary thyroid carcinoma through microarray meta analysis and quantification of its discriminatory power in independent validation

**DOI:** 10.1186/1755-8794-4-30

**Published:** 2011-04-06

**Authors:** Klemens Vierlinger, Markus H Mansfeld, Oskar Koperek, Christa Nöhammer, Klaus Kaserer, Friedrich Leisch

**Affiliations:** 1 Molecular Medicine, AIT - Austrian Institute of Technology, A-1190 Vienna, Austria; 2 Department of Clinical Pathology, University of Vienna Medical School, A-1090 Vienna, Austria; 3 Institute of Applied Statistics and Computing, University of Natural Ressources and Life Sciences, 1190 Vienna, Austria

## Abstract

**Background:**

Several DNA microarray based expression signatures for the different clinically relevant thyroid tumor entities have been described over the past few years. However, reproducibility of these signatures is generally low, mainly due to study biases, small sample sizes and the highly multivariate nature of microarrays. While there are new technologies available for a more accurate high throughput expression analysis, we show that there is still a lot of information to be gained from data deposited in public microarray databases. In this study we were aiming (1) to identify potential markers for papillary thyroid carcinomas through meta analysis of public microarray data and (2) to confirm these markers in an independent dataset using an independent technology.

**Methods:**

We adopted a meta analysis approach for four publicly available microarray datasets on papillary thyroid carcinoma (PTC) nodules versus nodular goitre (NG) from N2-frozen tissue. The methodology included merging of datasets, bias removal using distance weighted discrimination (DWD), feature selection/inference statistics, classification/crossvalidation and gene set enrichment analysis (GSEA). External Validation was performed on an independent dataset using an independent technology, quantitative RT-PCR (RT-qPCR) in our laboratory.

**Results:**

From meta analysis we identified one gene (SERPINA1) which identifies papillary thyroid carcinoma against benign nodules with 99% accuracy (n = 99, sensitivity = 0.98, specificity = 1, PPV = 1, NPV = 0.98). In the independent validation data, which included not only PTC and NG, but all major histological thyroid entities plus a few variants, SERPINA1 was again markedly up regulated (36-fold, p = 1:3*10^-10^) in PTC and identification of papillary carcinoma was possible with 93% accuracy (n = 82, sensitivity = 1, specificity = 0.90, PPV = 0.76, NPV = 1). We also show that the extracellular matrix pathway is strongly activated in the meta analysis data, suggesting an important role of tumor-stroma interaction in the carcinogenesis of papillary thyroid carcinoma.

**Conclusions:**

We show that valuable new information can be gained from meta analysis of existing microarray data deposited in public repositories. While single microarray studies rarely exhibit a sample number which allows robust feature selection, this can be achieved by combining published data using DWD. This approach is not only efficient, but also very cost-effective. Independent validation shows the validity of the results from this meta analysis and confirms SERPINA1 as a potent mRNA marker for PTC in a total (meta analysis plus validation) of 181 samples.

## Background

Thyroid nodules are endemic in iodine deficient areas, like Europes alpine regions, where they have a prevalence of 10-20%. They are classified by their histology into five main classes: the benign types Nodular Goiter (NG) and Follicular Thyroid Adenoma (FTA), and the malignant entities Follicular Thyroid Carcinoma (FTC), Papillary Thyroid Carcinoma (PTC), Medullary Thyroid Carcinoma (MTC) and Anaplastic Thyroid Carcinoma (ATC). Only approximately 5% - 10% of thyroid nodules are malignant [1], the majority of which are papillary carcinomas. Conventionally, discrimination between benign and malignant thyroid nodules is attempted by fine needle aspiration biopsy (FNAB) followed by cytological assessment. Despite many advances in the diagnosis and treatment of thyroid nodules and thyroid cancer, these methods have a well known low specificity [2], resulting in an 'indeterminate' or 'suspicious' diagnosis in 10% - 20% of cases. These patients usually undergo surgery, although in only 20% of these cases the nodules are actually malignant [3,4]. This leads to a number of patients unnecessarily treated for malignant disease.

In other types of cancer it has been shown that gene expression profiling can add substantial value to the discrimination of the different clinically relevant tumor-entities [5,6]. To date, many studies have tried to classify the different entities of thyroid carcinoma on the basis of their gene expression profiles. Each study has published a gene list which they believe discriminates between benign and malignant thyroid nodules or between different tumor entities. However, the lists have no or very few genes in common and applying a classifier from one study to the data from another study generally yields poor classification results. A notable exception to this are the studies from Jarzab et al. and Eszlinger et al. [7,8] who established a 20-gene signature for PTC which they were able to apply to another study and classify all samples correctly.

Here we focus on Papillary Thyroid Carcinoma (PTC), which is the most common and therefore most extensively studied form of thyroid cancer. We hypothesised, that feature selection based on a larger sample cohort will be more robust than using single studies, so we decided for a meta analysis approach which allows us to analyse all publicly avail-able datasets (99 samples) for PTC with one common analysis approach. From this dataset we were able to identify SERPINA1 as a single gene which allows to discriminate between PTC and benign nodules or healthy tissue with 99% accuracy (one misclassification). To validate these findings and to quantify the discriminatory power of SERPINA1 for the detection of PTC, we performed RT-qPCR experiments on an independent set of thyroid nodules (instantaneous sections) measuring the mRNA levels of SERPINA1. In contrast to the meta analysis data, we included not only PTC and benign nodules but also all other histological entities of thyroid nodules plus rare histological variants (follicular variant and tall cell PTC). Despite the marked overrepresentation of these difficult to diagnose histological variants, the classification accuracy was still as high as 93%.

Encouraged by the high classification accuracy in the independent validation data, showing the validity of our meta analysis approach, we went on to perform Gene Set Enrichment Analysis (GSEA) on the meta-analysis microarray data to elucidate some of the specific mechanisms involved in papillary thyroid tumorigenesis.

## Results

### Data Integration

**Figure 1:**
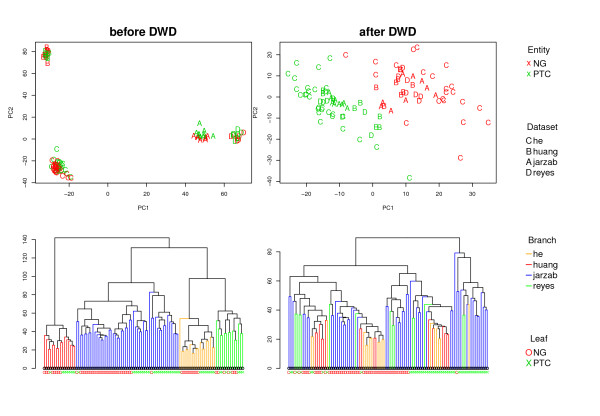
**DWD Integration**. The effect of DWD on the first two principal components (PC) and hierarchical clustering of the data. DWD was able to remove the separation between the datasets as indicated by the PC-plots and by the mixing of the branches in the dendrogram. The PC plots show that biological information is preserved after DWD integration (Samples cluster by dataset before integration and by tumor entity thereafter). Leaves in the dendrogram are colored by tumor entity and branches are colored by dataset.

Data Integration was performed using Distance Weighted Discrimination (DWD) [9]. Figure 1 shows the effect of DWD integration on the first two principal components (PC) and on hierarchical clustering. DWD removes the dataset bias very efficiently while preserving the biological information. Discretisation methods and bayesian methods were not able to remove the clustering by dataset in PCA or hierarchical clustering (data not shown).

### Classification

First, and as a quality measure for each study, each dataset was taken separately (before DWD-integration) and a pamr classification and leave-one-out cross-validation (loocv) was performed. The results of the cross-validations are near perfect with single samples classifying wrongly. However, with the exception of the classifier from the *He *dataset, none of these classifiers can be applied to any of the other datasets. Classification results are rarely ever higher than expected by chance. If, however, one uses the DWD-integrated data, the classifiers already fit much better (see Table 1).

**Table 1:** Classification Results before and after DWD integration

		before	DWD			after	DWD	
**test →** **train ↓**	**he**	**huang**	**jarzab**	**reyes**	**he**	**huang**	**jarzab**	**reyes**

he	1.00	1.00	0.98	1.00	1.00	1.00	0.96	1.00
huang	0.50	1.00	0.55	0.50	0.50	1.00	0.90	0.71
jarzab	0.50	0.81	1.00	0.57	0.89	1.00	1.00	1.00
reyes	0.78	0.50	0.92	1.00	0.89	0.88	0.90	1.00

Classification results for PTC-data when applying classifiers from one study on another study. Before (left) and after (right) DWD integration.

**Figure 2:**
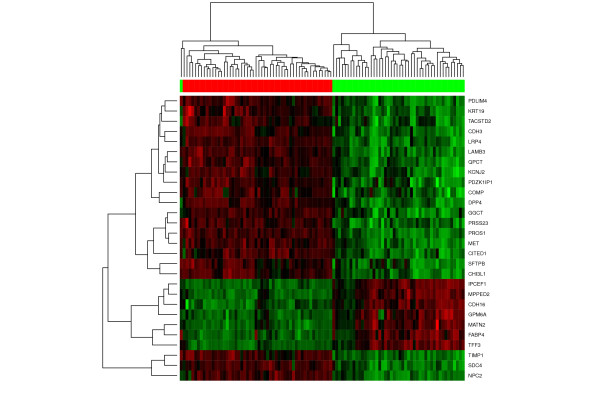
**Heatmap and hierarchical clustering of meta analysis data on further candidate marker genes**. As shown in the heatmap, there are a range of different genes with good discriminatory power between PTC and benign nodules. Therefore, removing SERPINA1 from the meta analysis dataset leads to a range of possible expression signatures with 99% classification accuracy in leave-one-out crossvalidation (see main text). In all cases, the same one sample is misclassified (see discussion for details). Columns correspond to samples and rows to genes, the red/green color bar on top of the heatmap corresponds to the histological classification (Red: benign, Green: PTC).

Then a pamr - classifier for the distinction of PTC versus various types of benign thyroid tissue was built for the complete DWD-integrated dataset and validated in a leave-one-out crossvalidation. This identified a one gene classifier, which classifies 99% of samples correctly in leave-one-out-crossvalidation (loocv). The discriminative gene is SERPINA1. If one removes the SERPINA1-probe from the analysis, pamr again finds a classifier with 99% accuracy in loocv, this time using a 9-gene signature. Removing these 9 genes yields another 9-gene classifier with a similar performance (99% accuracy), and further an 11-gene classifier with 99% accuracy. In all of these cases, the same sample is misclassified. The expression profile of these genes is visualised in the Figure 2.

### qPCR validation

**Figure 3:**
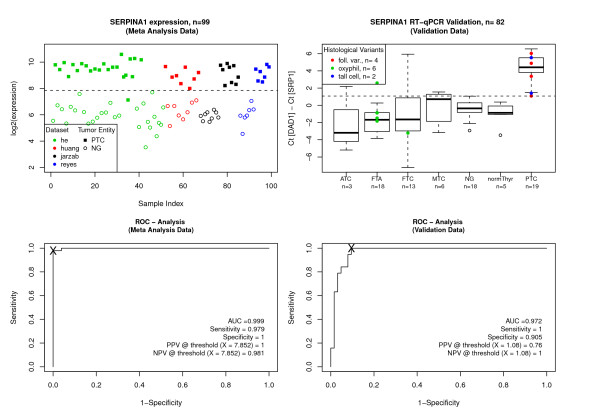
**SERPINA1 expression**. Expression values and receiver-operating-characteristics (ROC) analysis of the SERPINA1 gene in the meta analysis data (left) and the RT-qPCR independent validation data (right). Classification thresholds were chosen from ROC analysis (shown as 'X' in the ROC plots). Positive Predictive Values (PPV) were calculated as number of true positives/number of all positives, Negative Predictive Values (NPV) as number of true negatives/number of all negatives, both at the chosen threshold.

Next, we used RT-qPCR to test the discriminative power of SERPINA1 on an independent dataset generated in our own laboratories. Figure 3 shows the SERPINA1 gene expression and ROC analysis in the meta analysis across different studies and in the RT-qPCR data across the different entities. The upregulation of SERPINA1 in the meta analysis data is 8-fold (p *<*2 * 10^-16^) and 36-fold (p = 1.3 * 10^-10^) in the qPCR data (graphs are on a log2-scale). In the meta analysis data SERPINA1 expression accurately distinguishes PTC from benign nodules for 99% of the cases. Note that this is the DWD-integrated data. In the independent validation RT-qPCR data, where we picked samples from all histological entities, the papillary carcinomas again show a distinctly elevated expression of SERPINA1 compared to all other histological groups under investigation. A threshold value of 1.08 (SERPINA1 expression, normalised by DAD1-expression) was used as decision boundary for the decision PTC vs nonPTC. At this threshold, PTC can be detected with a sensitivity of 1 and a specificity of 0.905 (93% prediction accuracy). False Positive were 2 MTCs, 1 ATC, 2 FTCs (1 recurrent) and 1 FTA (oxyphilic). The meta analysis data classified one PTC falsely as benign, all others were correct.

### Differential Expression Analysis and Gene Set Enrichment

Encouraged by the high reproducibility of the meta analysis results in the external validation data, we went on to perform inference statistics on the meta analysis data. Table 2 shows the 20 most significant differentially expressed genes from inference statistics. SERPINA1 is the most differentially expressed gene, but there are many other genes which are also highly significant. Amongst the highly significant genes, most are upregulated. With a log2-ratio of 3.3 (corresponds to a 10-fold upregulation), SERPINA1 is not only the most significant gene, but also the gene with the largest effect size. We also used the results from inference statistics for GSEA with the goal of finding enriched gene sets, i.e. gene sets which have higher accumulated t-statistics than expected by chance [10]. In the integrated dataset there were four upregulated pathways (ECM-Receptor Interaction, Cell Adhesion, Cell Communication and p53 Signaling), three of which deal with extracellular processes and six downregulated pathways, all of which deal with metabolism and metabolite degradation. The same results were found when doing GSEA on each of the single studies separately.

**Table 2:** Differential Expression Analysis

SYMBOL	RefSeq	logFC	adj.P.Val	**Sens**.	**Spec**.	KEGGID
SERPINA1	NM_000295, NM_001002235, NM_001002236	3.30	7.81e-39	0.98	1.00	04610
PROS1	NM_000313	2.12	8.89e-34	0.98	1.00	04610
LRP4	NM_002334	2.80	8.89e-34	1.00	0.96	NA
NPC2	NM_006432	1.29	5.83e-33	1.00	0.94	04142
LAMB3	NM_000228, NM_001017402	2.46	5.11e-31	0.96	1.00	04510, 04512, 05200, 05222
DPP4	NM_001935	2.86	6.96e-31	0.98	0.98	NA
SDC4	NM_002999	1.68	5.50e-30	0.96	0.96	04512, 04514
IPCEF1	NM_015553	-2.10	2.88e-29	1.00	0.00	NA
QPCT	NM_012413	2.09	3.00e-29	0.98	1.00	NA
MPPED2	NM_001584	-2.28	7.73e-29	1.00	0.00	NA
TIMP1	NM_003254	1.85	1.48e-27	0.96	0.94	NA
TFF3	NM_003226	-3.42	2.07e-27	1.00	0.00	NA
PRSS23	NM_007173	1.48	2.32e-27	0.98	0.98	NA
MET	NM_000245	1.64	2.37e-27	0.94	0.98	04060, 04144, 04360, 04510, 04520, 05120, 05200, 05210, 05211, 05218
CDH3	NM_001793	2.72	3.45e-27	0.96	0.94	04514
GGCT	NM_024051	1.21	2.74e-26	0.98	0.92	00480
PDLIM4	NM_003687	2.21	3.37e-26	0.96	0.98	NA
KRT19	NM_002276	2.33	3.40e-25	0.98	0.98	NA
CITED1	NM_004143	3.17	4.42e-25	0.94	0.96	NA
CHI3L1	NM_001276	3.50	4.42e-25	0.96	0.90	NA

The first 20 entries in the toptable of differential expression in meta analysis data, including log2 fold changes (logFC) and Benjamini-Hochberg adjusted p-values (adj.P.Value) as calculated by the limma-software. Sensitivity and Specificity are given for the distinction of PTC vs NG at maximum accuracy. Annotational information like gene symbols, consensus sequence identifiers (RefSeq) and KEGG Pathway IDs are shown.

## Discussion

The present microarray meta analysis makes use of the latest methods for microarray data integration and classification. Nevertheless, meta-analysis of microarray data still poses a challenge, mainly because researchers ask at least partly different questions and hence use different experimental designs. Moreover, the number of thyroid tumor microarray data available to researchers to date is still comparably low (compared to breast cancer, e.g.). Therefore, when doing meta analysis one is forced to use all data available, even if the patient cohorts represent a rather heterogeneous and potentially biased population. More specifically, it is difficult to obtain a homogenous collection of control material (from healthy patients). These are usually taken from patients who were operated for other thyroid disease which is in turn very likely to cause a change in gene expression as measured on microarrays. In other studies, healthy tissue surrounding the nodule was takes as control material. The generation of homogeneous patient cohorts is further hampered by limited availability of patient data like age, gender, genetic background, etc.

Nevertheless we believe to have shown that there still is a great deal of information to be discovered in the wealth of DNA microarray data deposited in databases like ArrayExpress and GEO [11,12]. As shown in the present and other papers [9,13], methods for harvesting this information are available. Using approaches like DWD for data integration followed by classification and validation, it is possible to analyse a large number of samples at minimal cost. For the discovery of mRNA-based markers, a part of the scientific community has already moved on to other, more accurate, even higher parallel and even more expensive methods like mRNA sequencing using next-generation sequencing technologies. We (see Table 1) and others [14] have shown that feature selection in highly multivariate data like mircoarray data is not very robust, due to high feature numbers and low sample numbers. Applying these findings to next-generation sequencing data, it is likely that the feature selection problems will be even more pronounced, since feature numbers are even higher and even fewer people will be able to afford the analysis of sample sizes which account for the heterogeneous nature of clinical samples. This is why we believe that low cost/high sample number techniques like the one utilised here can provide valuable additional information.

This is also supported by another meta analysis on papillary thyroid carcinoma data which has been conducted by Fujarewicz et al. [15]. They used 3 datasets, two of which were used in our study as well (Jarzab and He). They skipped data integration and used a bootstrap strategy instead, which consisted of iterative construction of Support Vector Machine (SVM) classifiers (on features selected for each iteration) based on randomly selected sets of specimen and testing the classifiers on the remaining samples. Through this sampling scheme, they select genes which exhibit little study bias and good discrimination between tumor subtypes. They achieved 98.5% classification accuracy between PTC and benign nodules on a 20 gene classification rule.

When doing meta analysis of microarray data, researchers have also based their approach on modeling of inter-study variation [16] or on comparing gene lists from published studies. A meta analysis and meta review by Griffith et. al. [17] has summarised genes with a diagnostic potential in the context of thyroid disease. They published lists of genes which appeared in more than one high-throughput study (Microarray, SAGE) analysing thyroid disease and applied a ranking system. In their analysis SER-PINA1 scored the third highest. Six of our top 20 most significant genes appear in the list of Griffith et al. Their approach is very useful, as one can include all studies in the analysis and is not limited to the studies where raw data is available. However, the studies generally follow very different analysis strategies, some more rigorous than others. It is not under the control of the meta-analyst how the authors arrived at the gene lists. It is also not possible to assign a measure of confidence at the gene level. However, given the overlap in the marker genes identified by these two methods, they seem to complement each other well for in-silico marker discovery. ANOVA-based approaches are very useful for identifying differentially regulated genes but to our knowledge there is no method to test these genes in the meta analysis data for their discriminatory power.

Most of these lists were generated from microarray analysis. However, even when comparing the genes in the classifiers to gene lists generated with independent technologies, like cDNA library generation as performed by Kaserer et al. [18], there is substantial overlap. SERPINA1 appears in their lists as well as seven of our top 20 genes.

SERPINA1 has been reported before as a potential diagnostic marker for papillary thyroid carcinoma in a study investigating its protein expression in thyroid biopsy tissue by Immunocytochemistry and Western Blot [19]. In line with our results, they found SERPINA1 immunoreactivity in nine of ten papillary biopsies while the surrounding tissue showed no such reaction.

In GSEA, the most apparent feature of PTC is an upregulation of extracellular activities, like cell communication and adhesion and extracellular matrix receptor interactions. While the gene lists and the derived classification rules of the 4 studies under investigation here show little over-lap, we found the same pathways to be overrepresented when each of the single studies is being analysed separately. Upregulation of ECM microenvironment genes have been described as an effect of BRAF mutations through phospho-ERK1/2 signalling, which in turn may be involved in the up-regulation of some ECM remodeling genes like TSP-1 [20]. While ECM has been seen as mere scaffolding for many years, recently its role in tumor progression and invasion through ECM remodeling and stiffening of the tissue stroma became evident [21]. Similar Pathways have been reported to be implicated in the progression from simple ductal hyperplasia to atypical ductal hyperplasia and further to ductal carcinoma in situ in ER+ sporadic breast cancer by Emery et al. [22]. Like us, they found, amongst other pathways, overrepresentation of the ECM-receptor interaction, cell communication and p53 signaling pathways, but none of the downregulated metabolic pathways which we identified in papillary thyroid carcinoma. Their data also suggests, that in breast cancer progression, dysregulation of the ECM-epithelial cell interactions is an early event, even before any evidence of invasion becomes visible. Similarly, Birnie et al. [23] reported Cell Adhesion and ECM receptor interactions pathway dysregulated when comparing prostate cancer stem cells  to their normal and differentiated counterparts .

The power of the meta analysis approach adopted here is demonstrated by a 99% loocvaccuracy (97.9% weighted average accuracy in the study crossvalidation) for the distinction between papillary thyroid carcinoma and benign nodules. One sample was classified wrongly, and although it is not possible to correctly map the samples from this analysis to the original analysis [7], the misclassified sample is from the same group (PTC, validation group) as the sample which was wrongly classified in the original analysis. According to Jarzab et. al. the sample was an outlier because it contained only ≈ 20% tumor cells.

In the qPCR data we achieved a prediction accuracy of 93%. We chose the threshold to allow for false positives rather than false negatives. For this threshold 6 samples were false positives and none false negative. Three follicular nodules were false positive, one of them was a recurrent FTC nodule and one was an oxyphilic FTA nodule. The third was a classical FTC. Two MTC and one ATC nodules were falsely positive. The latter two entities are rather easy to distinguish by alternative methods like cytology or serum calcitonin testing (MTC). However, the raw, unnormalised data (not shown) suggests, that the slightly elevated levels in the MTC and ATC group may not be down to elevated SER-PINA1 levels, but to a different cellular turnover in these cells which leads to a problem in the normalisation procedure. A more thorough search for appropriate normalisation genes may circumvent this problem.

Gerhard et al. [24] studied the intra- and inter-observer variability in the cytological assessment of fine needle aspirates from thyroid nodules. They found 24% (n = 97) disagreement between two observers, both of them experienced pathologists. While most of these disagreements were between different follicular nodules, there was still an inter-observer disagreement of 6.3% for the PTC nodules. This highlights the need for objective diagnostic methods which include the possibility to assign a measure of confidence in the final diagnosis. While PTC shows the most distinct cytological features and is therefore relatively easy to diagnose, a molecular diagnostic test could also provide the possibility to include molecular markers for tumor entities which are harder to diagnose by cytology.

## Conclusions

These results are very encouraging, however, more studies need to be carried out in order to be able to transfer these findings into clinical applications for routine diagnostics. Firstly, markers with sufficient sensitivity and specificity for the other histological subtypes need to be found. There are few studies reporting molecular classifiers for follicular thyroid disease [25,26], and these classifiers cannot be applied to data from other studies (data not shown). To date, microarray studies for medullary and anaplastic thyroid carcinoma are largely missing. Secondly, in all of these studies, frozen thyroid nodules e.g. from instantaneous section pathology have been used. However, molecular markers for thyroid carcinomas will only be useful for routine diagnostics, if they exhibit the same discriminative power in fine needle aspiration biopsy (FNAB) samples. Microarray data from a study on thyroid FN-ABs [27] and a study on FNABs of various different tumor types [28] show good correlation between the aspirate and the tissue, but more detailed data is needed.

## Methods

### Datasets

Datasets were downloaded either from websites or from public repositories (GEO, ArrayExpress, see Table 3).

**Table 3:** Datasets used for meta analysis

	Published	PTC	**o.d**.	c.lat	Platform	Size
He	PNAS 2005	9	0	9	Affy U133plus	54k
Huang	PNAS 2001	8	0	8	Affy U95A	12k
Jarzab	Cancer Res 2005	23	11	17	Affy U133A	22k
Reyes	not published	7	0	7	Affy U133A	22k

Microarray Data used for meta analysis. The studies used 2 types of benign samples: samples from patients that were operated for other thyroid disease (o.d) and samples from the contralateral lobe (c.lat). Data was obtained from GEO http://www.ncbi.nlm.nih.gov/geo/ or institutional websites.

### Gene Mapping

The first step in any meta analysis of microarray data is to find the set of genes which is shared by all microarray platforms used in the analysis. Traditionally, overlap is assessed by finding common Entrez-Gene or UniGene (National Center for Biotechnology Information, http://www.ncbi.nlm.nih.gov/) identifiers. This, however, disregards all possible splice variations in the genes under investigation. For example, if a gene had 2 splice variants, one of which was Differentially expressed in the experiment and the other not and if one platform would contain an oligo specific only to the Differentially expressed variant and the other platform only an oligo to the other variant, then a matching based on UniGene would merge probes which measure different things.

To overcome this problem, the approach adopted here merges only probes which annotate to the same set of RefSeq identifiers. To this end all matching RefSeqs were downloaded for each probe(set) via the Bioconductor annotation packages (hgu133a, hgu95a and hgu133plus2; available at http://www.bioconductor.org). Probes which annotate to the same set of RefSeq identifiers were deemed to be similar. The median value was used, if one set of RefSeqs was represented by multiple probes on the array. 5707 different sets of RefSeqs were present on all arrays.

### Preprocessing and Data Integration

First each dataset was background-corrected and normalised separately, as recommended for each platform [29,30], then they were merged and quantile normalised collectively. Despite all preprocessing, it has been shown that data generated in different labs or on different microarray platforms or on different generations of the same platform may not be comparable due to platform or lab specific biases [8]. This is also evident from principal component analysis and hierarchical clustering of the merged data as shown in Figure 1. In order to correct for these biases, methods have been developed for integration of microarray data. One of these methods is Distance Weighted Discrimination (DWD) which is described in detail elsewhere [9]. Briefly, DWD projects data points onto the normal vector of a class (dataset) - separating hyperplane as calculated by a modified Support Vector Machine (SVM) and sub-tracts the class (dataset) means. Therefore, for a multi-class problem (more than 2 datasets to merge), the datasets need to be merged sequentially. For 4 datasets this leads to 24 different possibilities for merging, not including tree structured approaches, e.g instead of (((1 + 2) + 3) + 4), consider ((1 + 2) + (3 + 4)). The merging orders applied here were chosen on the general idea that similar and larger datasets should be merged first and more disparate ones later (personal communication). It is also worth noting, that adding a sample to a DWD merged dataset will change the whole dataset just like adding a new number to a vector of numbers will change its mean.

### Classification

For probe selection, classification and crossvalidation a nearest shrunken centroid method was chosen [31] (implemented in the Bioconductor package pamr). Briefly, it calculates several different possible classifiers using different shrinkage thresholds (i.e. different number of genes) and finds the best threshold from crossvalidation. Here we picked the classifier with the smallest number of genes (largest threshold), if more than one threshold yielded the same crossvalidation results.

### External Validation

Thyroid nodules were obtained from instantaneous section pathology at the Vienna General Hospital with the approval of the local ethics committee. We analysed 82 thyroid samples of 7 different entities: ATC(n = 3), FTA (n = 18), FTC (n = 13), MTC (n = 6), normal thyroid (n = 5), PTC (n = 19) and NG (n = 18). The choice of nodules also included a high rate of histological variants, like follicular variant PTC, tall cell PTC and oxyphilic follicular nodules. Approximately 30 mg of tissue was lysed in RLT-buffer on a FastPrep FP120 instrument (Qbiogene p/n 6001-120) and extracted using Qiagen All-Prep DNA/RNA extraction (Qiagen p/n 80204) according to manufacturers instructions. RNA quality was assessed on the Agilent 2100 Bioanalyser (p/n G2938C).

We had chosen following ABI TaqMan Assays: ACTB (Hs99999903_m1), CASC3 (Hs00201226_m1), DAD1 (Hs00154671_m1), PPIA (Hs99999904_m1) and SerpinA1 (Hs01097800_m1). Oligo-dT primed reverse transcription was done using Superscript III (Invitrogen), following the suppliers instructions. RT reaction with 180 ng RNA was used for three controls (ACTB, DAD1, PPIA) and the SerpinA1 gene in triplicates for quantitative PCR (12 reactions). We used 125 * μ*l of the ABI 2× Gene-Expression-Mastermix, 20 * μ*l cDNA and 42,5 * μ*l RNase free water for the PCR mastermix. Aliquots of 15 * μ*l (for 4 assays in triplicates) were added to a 1 * μ*l+4 * μ*l dilution of each assay (5 * μ*l diluted assay plus 15 * μ*l cDNA Mastermix). Cycling conditions were 2 sec 50°C, 10 sec 95°C and then 15 sec 95°C, 1 sec 60°C for 50 cycles (ABI 5700 Sequence Detection System). Using the method of Vandesompele et.al [32], DAD1 was picked as the gene with the highest stability and therefore used as housekeeping gene for normalisation.

### Gene Set Enrichment Analysis (GSEA)

First, inference statistics was calculated using the bioconductor package limma [33]. This includes non-specific filtering and linear modelling using empirical bayes moderated variances and Benjamini-Hochberg correction for multiple testing. For each gene, limma calculates a log odds ratio for differential expression (B-value). GSEA for pathway enrichment was performed on Students t-statistics using the geneSetTest function in the limma package. All data analysis except DWD-integration (Matlab^®^, Natick, MA) was done in R/bioconductor http://www.bioconductor.org[34]. R-codes and qPCR raw data is available at http://www.methcancerdb.net/methcancerdb/img/ThyroidMetaAnalysis.zip.

## Competing interests

The authors declare that they have no competing interests.

## Authors' contributions

KV, CN, KK and FL carried out the conception and design of the study, statistical analysis was carried out by KV under supervision of FL, conception and completion of molecular analysis was done by MM, collection and assessment of clinical material and data was done by KK and OK. KV prepared the manuscript including critical intellectual input and revisions by all authors. All authors read and approved the final manuscript.

## Pre-publication history

The pre-publication history for this paper can be accessed here:

http://www.biomedcentral.com/1755-8794/4/30/prepub
